# Expression of Wnt5a and ROR2, Components of the Noncanonical Wnt-Signaling Pathway, is Associated with Tumor Differentiation in Hepatocellular Carcinoma

**DOI:** 10.1245/s10434-023-14402-6

**Published:** 2023-10-09

**Authors:** Kazuki Wakizaka, Toshiya Kamiyama, Tatsuhiko Kakisaka, Tatsuya Orimo, Akihisa Nagatsu, Takeshi Aiyama, Shunsuke Shichi, Akinobu Taketomi

**Affiliations:** 1 Department of Gastroenterological Surgery I, Hokkaido University Graduate School of Medicine, Sapporo, Japan; 2 Department of Surgery, Seiwa Memorial Hospital, Sapporo, Japan

**Keywords:** Differentiation, Hepatocellular carcinoma, Immunohistochemistry, ROR2, Wnt signaling

## Abstract

**Background:**

Wnt5a is the key ligand of the noncanonical Wnt pathway, and receptor tyrosine kinase-like orphan receptor 2 (ROR2) is a receptor associated with Wnt5a. The association between the noncanonical Wnt-signaling pathway and carcinogenesis in hepatocellular carcinoma (HCC) is unclear. This study investigated the significance of ROR2 expression in HCC.

**Methods:**

The study examined ROR2 expression in liver cancer cell lines. Immunohistochemical staining of ROR2 was performed on 243 resected HCC specimens. The study investigated ROR2 expression and its association with clinicopathologic factors and prognosis.

**Results:**

Findings showed that ROR2 was expressed in well-differentiated Huh7 and HepG2 cells, but not in poorly differentiated HLE and HLF cells. Expression of ROR2 was positive in 147 (60.5%) and negative in 96 (39.5%) HCC specimens. A significant association was shown between ROR2 negativity and high alpha-fetoprotein (AFP) level (*P* = 0.006), poor differentiation (*P* = 0.015), and Wnt5a negativity (*P* = 0.024). The 5-year overall survival (OS) rate for the ROR2-negative group (64.2 %) tended to be worse than for the ROR2-positive group (73.8%), but the difference was not significant (*P* = 0.312). The 5-year OS rate was 78.7% for the ROR2+Wnt5a+ group, 71.3 % for the ROR2+Wnt5a− group, 80.8% for the ROR2−Wnt5a+ group, and 60.5 % for the ROR2−Wnt5a− group. The OS in the ROR2−Wnt5a− group was significantly poorer than in the ROR2+Wnt5a+ group (*P* = 0.030). The multivariate analysis showed that Wnt5a−ROR2− was an independent prognostic factor (hazard ratio, 2.058; 95% confidence interval, 1.013–4.180; *P* = 0.045).

**Conclusions:**

The combination of ROR2 and Wnt5a may be a prognostic indicator for HCC. The Wnt5a/ROR2 signal pathway may be involved in the differentiation of HCC. This pathway may be a new therapeutic target for HCC.

Hepatocellular carcinoma (HCC) is the fifth most commonly occurring cancer in the world.^1^ Liver resection is one of the most effective treatments for HCC. However, even after curative resection, HCC has a high recurrence rate.^2^ Better understanding of the mechanisms of HCC carcinogenesis and progression is critical to identification of novel target molecules for treatment and improvement of the prognosis for HCC patients.

The Wnt-signaling pathway is classified into canonical and noncanonical pathways. In the canonical Wnt-signaling pathway, β-catenin, which is normally degraded and maintained at low levels in the cytoplasm, accumulates in the cytoplasm from inhibition of degradation by Wnt stimulation. Accumulated β-catenin then translocates to the nucleus and binds to transcription factors to promote gene expression.^3^ Inappropriate activation of the canonical pathway is associated with carcinogenesis via abnormal accumulation of cytoplasmic β-catenin and its translocation to the nucleus.^4^ However, the association between the noncanonical pathway, which does not involve β-catenin activity, and carcinogenesis or tumor progression is not well known.

The Wnt family members function as ligands in the Wnt-signaling pathway, and the Wnt family has 19 members in mammals.^5^ Wnt5a is the key ligand of the noncanonical pathway.^6^ The noncanonical pathway is subclassified into the planar cell polarity (PCP) pathway and Ca^2+^ pathways.^7^ Through these pathways, Wnt5a signaling plays an important role in regulating cell differentiation, proliferation, migration, adhesion, and polarity.^6^ Our previous study showed that Wnt5a expression is a prognostic factor for HCC, and that overexpression of Wnt5a decreased proliferation and the epithelial-mesenchymal transformation promoters vimentin, SNAI2, MMP2, and MMP9, suggesting that Wnt5a may be a tumor suppressor in HCC.^8^

Several receptors for Wnt proteins that activate the noncanonical pathway have been identified, including frizzled family receptors (Frz), the receptor tyrosine kinase-like orphan receptor family (ROR), low-density lipoprotein receptor-related protein co-receptor (LRP), and receptor tyrosine kinase (Ryk).^6^ As the receptor for Wnt5a, ROR2 functions in the noncanonical pathway,^6,9^ and as a member of the tyrosine kinase superfamily, ROR2 is a co-receptor that forms a receptor complex with the Frz receptor.^10,11^

During early embryonic development, ROR2 is expressed in a variety of tissues and plays a key role in skeletal, neurologic, and midgut development.^12,13^ In adult tissues, the expression of ROR2 is repressed and limited to parathyroid, testicular, and uterine tissues.^14^ Findings show that ROR2 mediates several functions of the noncanonical Wnt-signaling pathway, including activation of the PCP pathway and the Ca^2+^ pathway.^7^

The relationship between cancer and ROR2 has been investigated, but the results have been unclear. Studies have shown that ROR2 is overexpressed in renal cancer, oral cancer, and malignant melanoma but suppressed in colon cancer.^15–18^ Thus, the role of ROR2 in cancer may differ depending on the type of cancer. Its role in carcinogenesis has not been completely determined.

The current study investigated the significance ROR2 expression and its association with Wnt5a in HCC.

## Methods

### Cell Lines

Human liver cancer cell lines HLE, HLF, HepG2, and Huh7 were obtained from the Japanese Collection of Research Bioresources Cell Bank (Osaka, Japan) and authenticated by short tandem repeat profiling. Cells were cultured in Dulbecco’s Modified Eagle’s Medium containing 10% fetal bovine serum at 37 °C in 5% carbon dioxice (CO_2_). The medium was replaced every second day.

### Western Blotting

Cells were cultured to reach 80% confluence and harvested using lysis buffer on ice. Equal amounts of protein (10 µg) were separated by sodium dodecyl sulfate–polyacrylamide gel electrophoresis and transferred to polyvinylidene difluoride membranes. After the membranes were blocked with 3 % bovine serum albumin, they were immunoblotted using primary antibodies against ROR2 (PAB3385, 1:1000; Abnova, Taipei, Taiwan), Wnt5a (ab174963, 1:500; Abcam, Cambridge, UK), and glyceraldehyde 3-phosphate dehydrogenase (GAPDH; #3683, 1:1000; Cell Signaling Technology, Inc., Danvers, MA, USA). The blots then were reacted with secondary anti-rabbit antibodies (#7074, 1:5000; Cell Signaling Technology, Inc.), followed by detection of bands with SuperSignal West Dura Extended Duration Substrate (34076; Thermo Fisher Scientific, Waltham, MA, USA).

### Invasion Assay

Invasion assays were performed in Matrigel invasion chambers (24 wells, 8 µm; 354480; Corning, Bedford, MA, USA). Cells (2.5 × 10^4^) in fetal bovine serum (FBS)-free medium were seeded in the upper chambers, and medium containing 10% FBS was added to the lower chambers. Cells above the membrane were wiped off using a cotton swab after 24 h. Membranes were stained and average values were obtained by counting five fields per membrane under a microscope (×10).

### Patients and Tissue Samples

We retrospectively screened 243 patients who underwent hepatic resection for HCC between January 1997 and December 2006 at our institution. After surgery, the patients were followed up by monitoring dynamic computed tomography and/or magnetic resonance imaging and tumor markers every 3 months on an outpatient basis. Combined examination of tumor markers and imaging studies was used to diagnose recurrence of HCC.

From the medical records of all the patients, we reviewed clinical information including sex, age, markers of hepatitis B virus, hepatitis C virus, serum albumin, serum α-fetoprotein (AFP), and protein induced by vitamin K absence or antagonist (PIVKA)II. We also reviewed tumor size, tumor number, vascular invasion, lymph node metastasis, and the pathologic findings of background liver. We used the criteria of the Liver Cancer Study Group of Japan (6th edition) to determine the tumor-node-metastasis stage of HCC.^19^

Informed consent of patients recruited between 1997 and 2000 was obtained in the form of opt-out on the website of Hokkaido University Hospital. Written informed consent was obtained from patients recruited between 2001 and 2006. This research was approved by the Institutional Review Board of Hokkaido University Hospital (017-0237) and performed in compliance with the Declaration of Helsinki.

### Immunohistochemical Staining

For immunohistochemical staining, 4-μm-thick sections of formalin-fixed and paraffin-embedded specimens were used. The sections were deparaffinized using xylene and ethanol, and antigen retrieval was performed by incubating sections in Target Retrieval Solution (pH 9.0; 415211; Nichirei Biosciences Inc., Tokyo, Japan) for 30 min at 95 °C. The samples were incubated with Block Ace (UKB80; KAC Co. Ltd., Kyoto, Japan) for 5 min to block nonspecific antibody reactions and incubated overnight at 4 °C with anti-ROR2 antibody (PAB3385, 1:500; Abnova) and anti‑Wnt5a antibody (LS‑C47384, 1:2000; LifeSpan BioSciences, Inc., Shirley, MA, USA). The samples were incubated in Histofine Simple Stain MAX PO (MULTI) (724152; Nichirei Biosciences) for 30 min at room temperature.

**Fig. 1:**
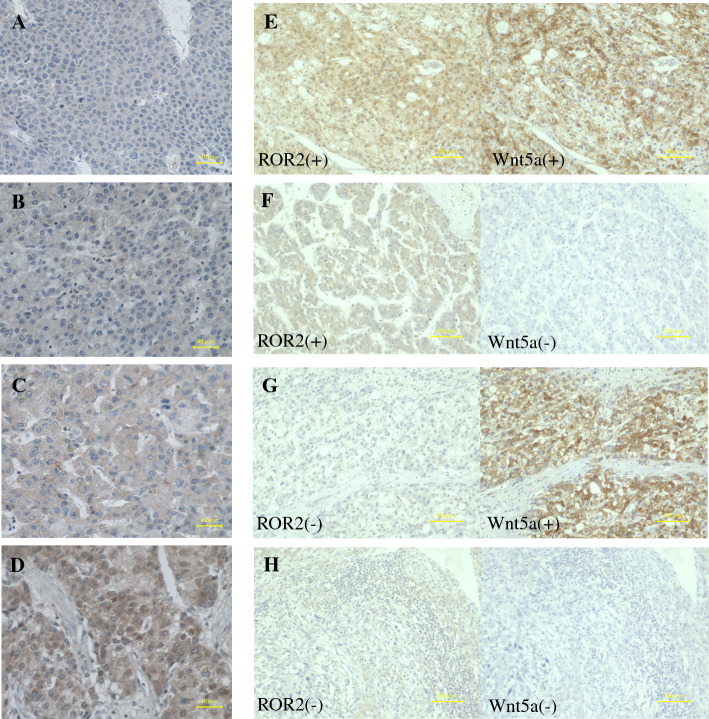
**A**–**D** Representative immunohistochemical staining of ROR2 in hepatocellular carcinoma tissues. Immunoreactivity was evaluated on the basis of cytoplasmic stain intensity: **A** score 0 (no staining), **B** score 1 (weak staining), **C** score 2 (positive staining), **D** score 3 (strong staining). A score of 2 or 3 indicated ROR2 positivity. **E**–**H** Representative images of four groups according to the expression status of ROR2 and Wnt5a: **E** ROR2+Wnt5a+, **F** ROR2+Wnt5a−, **G** ROR2−Wnt5a+, and **H** ROR2−Wnt5a–.

Staining was visualized using 3,3′ diaminobenzidine, and sections were counterstained with hematoxylin. Immunoreactivity was evaluated on the basis of cytoplasm staining intensity. The scores for staining intensity were as follows: 0 (no staining), 1 (weak staining), 2 (positive staining), and 3 (strong staining) (Fig. 1). A score of 2 or 3 indicated positive ROR2 staining. Wnt5a staining was evaluated as described in our previous report.^8^ Wnt5a-positive cells were defined according to the immunoreactivity on the cell membrane regardless of cytoplasmic immunoreactivity. The immunohistochemical staining pattern of Wnt5a in HCC was heterogeneous, and Wnt5a positivity was recorded if the proportion of Wnt5a-positive cells was more than 50%.

Four groups according to the expression status of ROR2 and Wnt5a (ROR2+Wnt5a+, ROR2+Wnt5a−, ROR2−Wnt5a+, and ROR2−Wnt5a− groups) also were examined (Fig. 1). Two authors blinded to the clinical and pathologic parameters evaluated the results of immunohistochemical staining.

### Statistical Analysis

Statistical analysis was performed using EZR version 1.35 (Saitama Medical Center, Jichi Medical University, Saitama, Japan).^20^ Fisher’s exact test was used to examine the correlation between ROR2 expression and clinical and pathologic variables. The Kaplan–Meier method was used to plot overall survival (OS) and relapse-free survival (RFS) curves, and the curves were compared using the log-rank test. The Cox proportional hazards regression model was used to perform multivariate analyses. A *P* value lower than 0.05 was considered statistically significant.

## Results

### ROR2 Expression in Liver Cancer Cell Lines

**Fig. 2:**
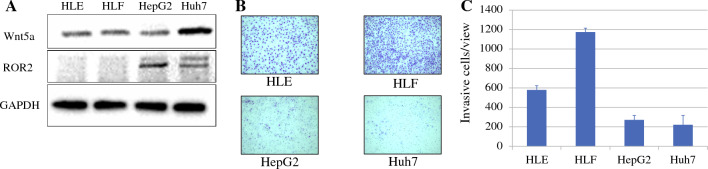
**A** Western blotting of ROR2 in liver cancer cell lines. Wnt5a expression was high in Huh7 cells. ROR2 was expressed in Huh7 and HepG2 cells, which are well-differentiated cell lines, but not in HLE and HLF cells, which are poorly differentiated cell lines. **B**, **C** Comparison of invasion ability of HCC cell lines. **B** Staining of the membrane in invasion assays using the indicated cell lines (×10). **C** The poorly differentiated cell lines HLE and HLF tended to have higher invasion ability than the well-differentiated cell lines HepG2 and Huh7.

We examined the expression of ROR2 in liver cancer cell lines with different degrees of differentiation using Western blotting. We chose HLE and HLF as poorly differentiated cell lines and Huh7 and HepG2 as well-differentiated cell lines.^21^ Wnt5a expression tended to be high in the well-differentiated cell lines, especially in Huh7 cells. As shown in Fig. 2A). ROR2 was expressed in the Huh7 and HepG2 well-differentiated cell lines, but not in the HLE and HLF poorly differentiated cell lines. In invasion assays, the poorly differentiated cell lines HLE and HLF tended to have higher invasion ability than the well-differentiated cell lines HepG2 and Huh7 (Fig. 2B, C).

### Clinical and Pathologic Characteristics and ROR2 Expression in HCC Patients

Our analysis using liver cancer cell lines showed that ROR2 expression may be related to tumor differentiation in HCC. To further explore the relationship between ROR2 expression and tumor differentiation, we examined clinical samples. The clinical and pathologic characteristics of the HCC patients included in this study are summarized in Table 1. The expression of ROR2 in HCC tumor samples was positive in 147 patients (60.5%) and negative in 96 patients (39.5%).

**Table 1:** Clinicopathologic characteristics of patients

Characteristics	No. of patients (%)
*Sex*	
Male	200 (82.3)
Female	43 (17.7)
Median age: years (range)	63 (35–82)
*Viral infection*	
HBV	90 (37.0)
HCV	92 (37.9)
HBV + HCV	8 (3.3)
NBNC	53 (21.8)
*Child-Pugh class*	
A	237 (97.5)
B	6 (2.5)
*Albumin (g/dl)*	
< 4	96 (39.5)
≥ 4	147 (60.5)
*AFP (ng/ml)*	
≤ 10	127 (52.3)
> 10	114 (46.9)
*PIVKAII (mAU/ml)*	
≤ 40	101 (43.2)
> 40	136 (56.0)
*Differentiation*	
Well	40 (16.5)
Moderate	156 (64.2)
Poor	47 (19.3)
*Tumor number*	
One	187 (77.0)
Multiple	56 (23.0)
*Tumor size (cm)*	
≤ 2	36 (14.8)
> 2 to ≤5	129 (53.1)
> 5 to ≤10	59 (24.3)
> 10	19 (7.8)
*Vascular invasion*	
Positive	39 (16.1)
Negative	204 (83.9)
*Lymph node metastasis*	
Positive	0 (0.0)
Negative	243 (100.0)
*pStage*^*a*^	
I	24 (9.9)
II	136 (56.0)
III	83 (34.2)
IVA	0 (0.0)
IVB	0 (0.0)
*Non-cancerous liver*	
No cirrhosis	157 (64.6)
Cirrhosis	86 (35.4)
*Wnt5a*	
Positive	63 (25.9)
Negative	180 (74.1)
*ROR2*	
Positive	147 (60.5)
Negative	96 (39.5)

*HBV* hepatitis B virus, *HCV* hepatitis C virus, *NBNC* non-hepatitis B virus and non-hepatitis C virus, *AFP* α-fetoprotein, *PIVKAII* protein induced by vitamin K absence or antagonist II. Some categories did not include 243 patients because of incomplete data.

^a^Liver Cancer Study Group of Japan, 6th edition

The correlations of clinical and pathologic characteristics with ROR2 expression are presented in Table 2, which shows that ROR2 negativity was significantly associated with a high level of AFP (*P* = 0.006) and poor tumor differentiation (*P* = 0.015). Expression of ROR2 was associated with Wnt5a expression (*P* = 0.024).

**Table 2:** Correlation between ROR2 expression and clinicopathologic characteristics

	ROR2 expression		*P* value
	Negative (*n* = 96)	Positive (*n* = 147)	
*Sex*			
Male	74	126	0.089
Female	22	21	
*Age (years)*			
≤ 60	40	62	1.000
> 60	56	85	
*HBV*			
Negative	56	89	0.789
Positive	40	58	
HCV			
Negative	52	91	0.234
Positive	44	56	
*AFP (ng/ml)*			
≤ 10	39	88	0.006^a^
> 10	55	59	
*PIVKAII (mAU/ml)*			
≤ 40	41	60	0.893
> 40	53	83	
*Differentiation*			
Well-moderate	69	125	0.015^a^
Poor	27	22	
*Tumor number*			
One	75	112	0.756
Multiple	21	35	
*Tumor size (cm)*			
≤ 5	64	101	0.779
> 5	32	46	
*Vascular invasion*			
Negative	80	124	0.859
Positive	16	23	
*Non-cancerous liver*			
No cirrhosis	62	95	1.000
Cirrhosis	34	52	
*Wnt5a*			
Negative	79	101	0.024^a^
Positive	17	46	

*HBV* hepatitis B virus, *HCV* hepatitis C virus, *AFP* α-fetoprotein, *PIVKAII* protein induced by vitamin K absence or antagonist II

^a^*P* < 0.05

### Survival Analysis

**Fig. 3:**
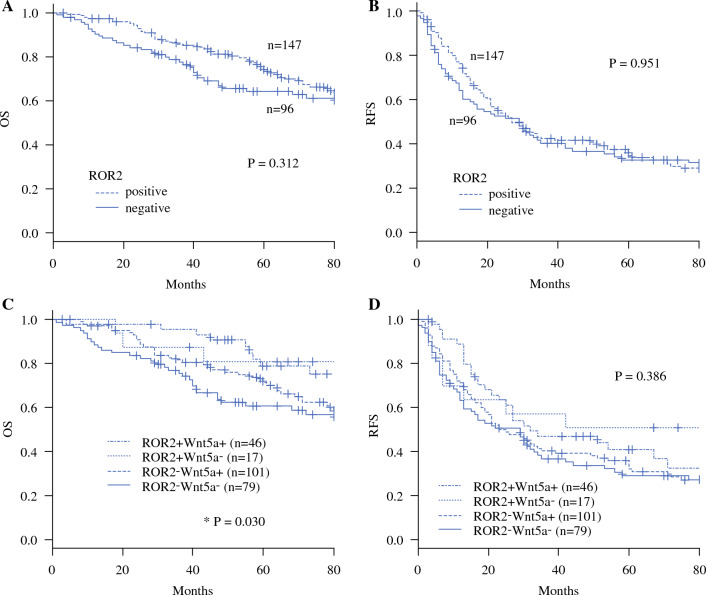
Survival analysis. **A** Overall survival (OS) and **B** relapse-free survival (RFS) curves were analyzed using the Kaplan–Meier method. The OS in the ROR2-negative group tended to be poorer than in the ROR2-positive group, but the difference was not significant (*P* = 0.312), and the RFS did not differ significantly between the groups (*P* = 0.951). Kaplan–Meier analysis of **C** OS and **D** RFS of four groups on the basis of the expression of ROR2 and Wnt5a showed that OS in the ROR2−Wnt5a− group was significantly poorer than in the ROR2+Wnt5a+ group (*P* = 0.030) and that RFS did not differ significantly between the groups (*P* = 0.386).

The 5-year OS rate was 73.8% in the ROR2-positive group and 64.2% in the ROR2-negative group (Fig. 3A). The OS in the ROR2-negative group tended to be poorer than in the ROR2-positive group, but the difference between the groups was not significant (*P* = 0.312). The 5-year RFS rate was 34.7% in the ROR2-positive group and 32.8% in the ROR2-negative group (Fig. 3B). The RFS did not differ significantly between the groups.

### Analyses of Four Groups on the Basis of ROR2 and Wnt5a Expression

We next performed analyses of four groups on the basis of ROR2 and Wnt5a expression. The correlations of clinical and pathologic characteristics with ROR2/Wn5a expression are presented in Table 3, which shows that ROR2−Wnt5a− was significantly associated with a high level of AFP (*P* = 0.006) and poor tumor differentiation (*P* = 0.001). The 5-year OS rate was 78.7 % in the ROR2+Wnt5a+ group, 71.3 % in the ROR2+Wnt5a− group, 80.8% in the ROR2−Wnt5a+ group, and 60.5% in the ROR2−Wnt5a− group (Fig. 3C). The OS in the ROR2–Wnt5a– group was significantly poorer than in the ROR2+Wnt5a+ group (*P* = 0.030).

**Table 3:** Correlation of ROR2 and Wnt5a expression with clinicopathologic characteristics

	ROR2 and Wnt5a expression				*P* value
	ROR2+Wnt5a+ (*n* = 46)	ROR2+Wnt5a– (*n* = 17)	ROR2-Wnt5a+ (*n* = 101)	ROR2-Wnt5a– (*n* = 79)	
*Sex*					
Male	41	14	85	60	0.272
Female	5	3	16	19	
*Age (years)*					
≤ 60	20	9	42	31	0.770
> 60	26	8	59	48	
*HBV*					
Negative	26	9	63	47	0.845
Positive	20	8	38	32	
*HCV*					
Negative	34	12	57	40	0.052
Positive	12	5	44	39	
*AFP (ng/ml)*					
≤ 10	29	11	59	28	0.006^a^
> 10	17	6	42	49	
*PIVKAII (mAU/ml)*					
≤ 40	17	7	43	34	0.940
> 40	27	10	56	43	
*Differentiation*					
Well-moderate	43	16	82	53	0.001^a^
Poor	3	1	19	26	
*Tumor number*					
One	34	12	78	63	0.806
Multiple	12	5	23	16	
*Tumor size (cm)*					
≤ 5	31	9	70	55	0.583
> 5	15	8	31	24	
*Vascular invasion*					
Negative	43	15	81	65	0.208
Positive	3	2	20	14	
*Non-cancerous liver*					
No cirrhosis	30	9	65	53	0.705
Cirrhosis	16	8	36	26	

*HBV* hepatitis B virus, *HCV* hepatitis C virus, *AFP* α-fetoprotein, *PIVKAII* protein induced by vitamin K absence or antagonist II

^a^*P* < 0.05

The 5-year RFS rate was 40.6% in the ROR2+Wnt5a+ group, 32.1% in the ROR2+Wnt5a− group, 50.7% in the ROR2–Wnt5a+ group, and 28.9% in the ROR2−Wnt5a− group (Fig. 3D). Th RFS did not differ significantly between the groups.

The significant prognostic factors in the univariate analysis were serum albumin (*P* = 0.005), serum AFP (*P* = 0.018), serum PIVKAII (*P* = 0.049), tumor number (*P* = 0.013), tumor size (*P* = 0.001), vascular invasion (*P* < 0.001), liver cirrhosis (*P* = 0.012), and Wnt5a/ROR2 expression (Wnt5a–ROR2− vs Wnt5a+ROR2+; *P* = 0.035). In the multivariate analysis, the independent prognostic factors in HCC patients were serum albumin (hazard ratio [HR] 1.565; 95 % confidence interval [CI] 1.000–2.452; *P* = 0.050), tumor number (HR, 1.931; 95 % CI 1.170–3.186; *P* = 0.010), tumor size (HR, 1.854; 95 % CI 1.114–3.085; *P* = 0.017), vascular invasion (HR 2.291; 95 % CI 1.340–3.915; *P* = 0.002), liver cirrhosis (HR 1.755; 95 % CI 1.116–2.761; *P* = 0.014), and Wnt5a/ROR2 expression (Wnt5a−ROR2− vs Wnt5a+ROR2+: HR 2.058; 95 % CI 1.013–4.180; *P* = 0.045) (Table 4).

**Table 4:** Uni- and multivariate analyses of prognostic factors for overall survival

	Univariate analysis			Multivariate analysis		
	HR	95 % CI	*P* value	HR	95 % CI	*P* value
Sex (male vs female)	1.030	0.581–1.823	0.921			
Age (> 60 vs ≤ 60 years)	1.426	0.926–2.196	0.107			
HBV (positive vs negative)	0.873	0.569–1.339	0.533			
HCV (positive vs negative)	1.132	0.743–1.724	0.565			
Albumin (< 4 vs ≥ 4 g/dl)	1.826	1.204–2.770	0.005^a^	1.565	1.000–2.452	0.050^a^
AFP (> 10 vs ≤ 10 ng/ml)	1.668	1.090–2.553	0.018^a^	1.416	0.899–2.227	0.133
PIVKAII (> 40 vs ≤ 40 mAU/ml)	1.570	1.002–2.459	0.049^a^	1.445	0.867–2.408	0.157
Tumor number (multiple vs one)	1.824	1.134–2.932	0.013^a^	1.931	1.170–3.186	0.010^a^
Tumor size (> 5 vs ≤ 5 cm)	2.110	1.384–3.218	0.001^a^	1.854	1.114–3.085	0.017^a^
Vascular invasion (positive vs. negative)	2.630	1.645–4.205	< 0.001^a^	2.291	1.340–3.915	0.002^a^
Differentiation (poor vs well-moderate)	1.361	0.826–2.241	0.226			
Non-cancerous liver (cirrhosis vs. no cirrhosis)	1.721	1.130–2.623	0.012^a^	1.755	1.116–2.761	0.014^a^
Wnt5a−ROR2−						
vs Wnt5a−ROR2+	1.249	0.790–1.974	0.339	1.264	0.770–2.077	0.353
vs Wnt5a+ROR2−	2.258	0.877–9.310	0.081	2.869	0.855–9.624	0.087
vs Wnt5a+ROR2+	2.028	1.049–3.918	0.035^a^	2.058	1.013–4.180	0.045^a^

*HR* hazard ratio, *CI* confidence interval, *HBV* hepatitis B virus, *HCV* hepatitis C virus, *NBNC* non-hepatitis B virus and non-hepatitis C virus, *AFP* α-fetoprotein, *PIVKAII* protein induced by vitamin K absence or antagonist II

^a^*P* < 0.05

## Discussion

This study demonstrated that ROR2 negativity determined by immunohistochemical staining was significantly associated with high AFP and low tumor differentiation in HCC. The OS in the ROR2-negative group tended to be worse than in the ROR2-positive group.

We then performed analysis of survival in four groups with various ROR2 and Wnt5a expressions. The 5-year OS rate was 78.7% for the ROR2+Wnt5a+ group and 60.5% for the ROR2−Wnt5a− group, and the difference was significant (*P* = 0.030). The multivariate analysis showed that Wnt5a−ROR2− was an independent prognostic factor for OS in HCC patients. Western blotting showed that ROR2 was not expressed in the poorly differentiated HCC cell lines. Together, these data suggest that the Wnt5a/ROR2-signaling pathway may be involved in HCC differentiation.

In this study, the HCC cases with ROR2 negativity tended to have a poor prognosis, but the difference was not significant. Expression of ROR2 was not shown to be a prognostic factor, but it may be an indicator in combination with Wnt5a expression. The relationship between ROR2 expression and tumor differentiation was shown in both clinical specimens and cell lines, in which decreased expression of ROR2 was associated with decreased tumor differentiation. These results suggest that ROR2 alone is weak in action but more potent when coexisting with the ligand Wnt5a, and that the Wnt5a/ROR2-signaling pathway may be involved in HCC differentiation.

As shown in the supportive data of our previous study, Wnt5a/ROR2-binding is associated with epithelial-mesenchymal transformation because the expression of E-cadherin decreased when the ligand Wnt5a was downregulated in Huh7, in which Wnt5a and ROR2 are co-expressed.^8^ The same phenomenon would be expected to occur when the expression of the receptor ROR2 is decreased, although the experiment should have been performed in which ROR2 expression was directly decreased.

A stepwise dedifferentiation occurs during the carcinogenesis and progression of HCC. Dysplastic nodules arise from regenerative nodules of the cirrhotic liver, which gradually develop into well-differentiated, moderately differentiated, and poorly differentiated HCC.^22^ Wnt/β-catenin-signaling, TGF- β/SMAD-signaling, and PI3K/AKT-signaling are widely activated in HCC and cooperatively promote dedifferentiation of HCC.^23^ Furthermore, some reports have shown that the noncanonical Wnt pathway inhibits the canonical Wnt pathway in HCC cell lines.^24^ Therefore, these studies combined with the result in the current study that ROR2 expression is decreased in poorly differentiated cell lines suggest that inactivation of the noncanonical pathway from decreased ROR2 expression may activate the canonical pathway, leading to dedifferentiation.

As further support, in our immunohistochemical staining of ROR2 in 243 HCC cases, ROR2 negativity was significantly associated with high level of AFP (*P* = 0.006) and poor differentiation (*P* = 0.015), which correlated with tumor differentiation. The association of the noncanonical pathway involving ROR2 with the aforementioned signaling involved in dedifferentiation should be investigated in future studies.

This study had several limitations. First, it was a retrospective single-center study. Therefore, prospective, multicenter studies are necessary to validate our results. To clarify the relationship between ROR2 and tumor differentiation in HCC, it is necessary to examine the signal transduction pathways involved.

In conclusion, our results showed that ROR2 in combination with Wnt5a may be a prognostic indicator in HCC. The Wnt5a/ROR2 signal pathway may be involved in the differentiation of HCC. Furthermore, this pathway may be a new therapeutic target for HCC.
